# Preliminary Education and Medical Colleges

**Published:** 1859-01

**Authors:** 


### EDITORIAL.

### PRELIMINARY EDUCATION AND MEDICAL COLLEGES.

In a recent number of the *Journal*, we copied a part of the published proceedings of the Board of Regents of the University of Michigan, in relation to the adoption of a standard of preliminary education to be required for admission into the medical department of that university. It appeared that the adoption of the proposed standard was actively opposed and defeated by two members of the medical faculty. This opposition to a very important measure, from men who hold salaried professorships in a state institution richly endowed, and one of whom had, for several years, industriously spread the impression that the exaction of a high standard of preliminary requirements constituted one of the chief merits of the institution, took many, even of their own friends, by surprise.

Some of the medical periodicals also noticed the action of the professors with decided disapprobation. This called forth from the gentlemen concerned, Professors Palmer and Sager, a letter in defence of their action, which was published in the *American Medical Gazette.* In this letter they seek to justify themselves by raising three leading objections to the proposed standard of requirements, as follows:

*First.* It requires each student, before admission to the medical department, to possess a certain amount of knowledge concerning the Greek and Latin languages. This, the learned professors seemed to think, was, at this age of the world, unnecessary.

*Second.* The possession of the proposed amount of preliminary education was to be ascertained, and certified to, by competent persons *not connected* with the *medical* department of the university.

This objection is certainly a singular one to emanate from members of the medical faculty. Inasmuch as the provision objected to relieves them entirely from the loss of time which a preliminary examination would require, and also from the responsibility of making it, one would suppose that it was shaped designedly for their satisfaction. And yet Professors Palmer and Sager really urge this as the chief ground of objection; not, however, because they seem to entertain any fear that other parties would be careless and unfaithful in making the required examinations. On the contrary, it is plainly evident from their letter that they fear the examination conducted by those having no place in the medical department would be *faithful*, and therefore so much dreaded as to deter large numbers from coming to the school; while if it were left entirely in the hands of the medical faculty it could be made as *nominal* as the ignorance of the student might require. In other words, the letter of Professors Palmer and Sager plainly implies that the medical department of their university would be quite willing to receive the credit abroad which a provision for exacting a high standard of preliminary education would give it, provided the examinations for determining such standard were to be conducted by the faculty alone, so as to enable them *practically* to admit whoever might apply. Comment upon such a position is unnecessary.

The *third* objection urged in the letter is, that the exaction of such a standard as was proposed would exclude a large proportion of those students who would otherwise attend, and therefore greatly diminish the number of students in the medical department of the university. To cover the selfishness of this objection, the professors become suddenly *democratic*, and gravely claim that the university being a state institution with ample pecuniary endowment, the people of the state have a right to its advantages for their intellectual culture; and, of course, the medical students of the state must have access, by right, to the medical department: therefore, any provision or standard of preliminary qualifications, which would restrict this right of access, would be unjust to the profession of the state. It requires but one or two plain questions to show the extreme absurdity of the position.

If a young man, who had never attended the primary schools sufficient to gain a competent knowledge of reading, writing, spelling, and arithmetic, should apply to Chancellor Tappen for admission to the literary department of the university, and propose to enter at once upon the course of study required for the degree of A.B., would he have a right to admission merely because the university is a *state* institution? Of course not. And pray tell us, by what process the same ignoramus can have any more *right* to admission into the *medical* department, and be put on a course of study for the dignified title of M.D.? It is undoubtedly true that the students of Michigan, whether literary or medical, have a right to admission into her university; but only on condition that they *qualify themselves* to profit by its several courses of instruction. To claim admission without such qualifications, would be as absurd as for the idle loafer to assert his right to a portion of the products of the soil without bestowing any of the labor necessary for their production. Such are the lame and impotent objections urged by Professors Palmer and Sager against the adoption of a just and appropriate standard of preliminary education for admission into the medical department of their state. We agree with the editors of the Cincinnati *Lancet and Observer*, that the position thus assumed by those representing the only independently endowed and salaried medical school in the North-west, is unworthy of themselves and highly derogatory to the interests of the whole profession. That an improvement in the preliminary education of medical students is a *sine qua non*, as a foundation for elevating the character and usefulness of the whole profession, is apparent to every intelligent man among us. And if a faculty, possessing the pecuniary independence of that connected with the Michigan school, would cheerfully and faithfully second the proposition made by their Board of Regents on this subject, they would speedily induce efficient action in the same direction in the several state and local medical associations. This again would react upon the faculties of other schools, and we should soon have a sure basis for improvement in the whole system of medical education.

But so long as a miserable ambition to swell the *mere number* of their classes induces them to deliberately block the wheels of advancement, their influence tends strongly to paralyze the efforts of all around them. While on this subject, we would call the attention of the profession to the resolutions adopted, almost unanimously, at the first meeting of the American Medical Association, in 1847. Will not the several state and local medical societies in the North-west take them into serious consideration at their next regular meetings?

They are as follows:

"*Resolved*, That this Convention earnestly recommends to the members of the medical profession throughout the United States, to satisfy themselves, either by personal inquiry or written certificate of competent persons, before receiving young men into their offices as students, that they are of good moral character, and that they have acquired a good English education, a knowledge of natural philosophy and the elementary mathematical sciences, including geometry and algebra, and such an acquaintance, at least, with the Latin and Greek languages as will enable them to appreciate the technical language of medicine, and read and write prescriptions.

"*Resolved*, That this Convention also recommends to the members of the medical profession of the United States, when they have satisfied themselves that a young man possesses the qualifications specified in the preceding resolution, to give him a written certificate stating that fact, and recording also the date of his admission as a medical student, to be carried with him as a warrant for his reception into the medical college in which he may intend to pursue his studies.

"*Resolved*, That all the medical colleges in the United States be, and they are hereby recommended and requested to require such a certificate of every student of medicine applying for matriculation; and when publishing their annual lists of graduates, to accompany the name of the graduate with the name and residence of his preceptor, the name of the latter being clearly and distinctly presented as certifying to the qualification of preliminary education."

